# Genetic Architecture of Feeding Behavior and Feed Efficiency in a Duroc Pig Population

**DOI:** 10.3389/fgene.2018.00220

**Published:** 2018-06-19

**Authors:** Rongrong Ding, Ming Yang, Xingwang Wang, Jianping Quan, Zhanwei Zhuang, Shenping Zhou, Shaoyun Li, Zheng Xu, Enqin Zheng, Gengyuan Cai, Dewu Liu, Wen Huang, Jie Yang, Zhenfang Wu

**Affiliations:** ^1^College of Animal Science and National Engineering Research Center for Breeding Swine Industry, South China Agricultural University, Guangdong, China; ^2^National Engineering Research Center for Breeding Swine Industry, Guangdong Wens Foodstuffs Group, Co., Ltd., Guangdong, China; ^3^Department of Animal Science, Michigan State University, East Lansing, MI, United States

**Keywords:** pigs, Duroc, GWAS, feed conversion ratio, residual feed intake, feeding behavior, feed efficiency

## Abstract

Increasing feed efficiency is a major goal of breeders as it can reduce production cost and energy consumption. However, the genetic architecture of feeding behavior and feed efficiency traits remains elusive. To investigate the genetic architecture of feed efficiency in pigs, three feeding behavior traits (daily feed intake, number of daily visits to feeder, and duration of each visit) and two feed efficiency traits (feed conversion ratio and residual feed intake) were considered. We performed genome-wide association studies (GWASs) of the five traits using a population of 1,008 Duroc pigs genotyped with an Illumina Porcine SNP50K BeadChip. A total of 9 genome-wide (*P* < 1.54E-06) and 35 suggestive (*P* < 3.08E-05) single nucleotide polymorphisms (SNPs) were detected. Two pleiotropic quantitative trait loci (QTLs) on SSC 1 and SSC 7 were found to affect more than one trait. Markers WU_10.2_7_18377044 and DRGA0001676 are two key SNPs for these two pleiotropic QTLs. Marker WU_10.2_7_18377044 on SSC 7 contributed 2.16 and 2.37% of the observed phenotypic variance for DFI and RFI, respectively. The other SNP DRGA0001676 on SSC 1 explained 3.22 and 5.46% of the observed phenotypic variance for FCR and RFI, respectively. Finally, functions of candidate genes and gene set enrichment analysis indicate that most of the significant pathways are associated with hormonal and digestive gland secretion during feeding. This study advances our understanding of the genetic mechanisms of feeding behavior and feed efficiency traits and provide an opportunity for increasing feeding efficiency using marker-assisted selection or genomic selection in pigs.

## Introduction

Pork is an important meat source for humans, accounting for nearly 40% of all meat consumed by the world population (Wang et al., 2015). The share of feed cost, which is the highest of the total production cost, remains high, ranging from 50 to 85% (Young et al., 2011). The key to reducing feed cost is to increase feed efficiency. Increasing feed efficiency not only reduces feed consumption while decreasing farming cost and energy use, but also lowers manure production and the total amount of potential greenhouse gas emission (O’Shea et al., 2012; Bartos et al., 2016).

In animal breeding programs, feed efficiency traits are difficult to improve by direct selection because feed efficiency cannot be measured directly (Hoque and Suzuki, 2008). Feed conversion ratio (FCR) and residual feed intake (RFI) are two traits that have been used to evaluate feed efficiency (Lu et al., 2017). FCR (the ratio of feed intake to output) is widely used to estimate feed efficiency in pig breeding because of its simplicity in calculation and its correlation with growth rate and body weight (BW) (Fan et al., 2010; Do et al., 2013a). However, previous genetic studies also show that FCR is not always effective (Webb and King, 1983). For example, pigs with low feed intakes and undesirably low gains may also have high FCRs (Webb and King, 1983; Iwaisaki, 1989; Smith et al., 1991). RFI is defined as the difference of feed intake between the actual feed eaten and the expected feed intake required for production and maintenance (Koch et al., 1963). RFI appears to be a better indicator of feed efficiency as compared to FCR. However, computation of RFI varies widely, depending on the predicted feed requirement for production and maintenance (Hoque et al., 2009; Do et al., 2013a, 2014), which adds to the difficulty of comparing different studies.

Feeding behavior is one of the most important factors affecting feed efficiency. Several studies have shown a strong genetic and phenotypic correlation between feeding behavior and feed efficiency traits in swine (Hoque et al., 2009). For instance, daily feed intake (DFI) has a positive genetic and phenotypic correlation with FCR (0.65 and 0.67, respectively) and RFI (0.95 and 0.90, respectively) (Do et al., 2013a). Therefore, to understand the molecular mechanism and genetic basis of feed efficiency, it is likely helpful to consider both feeding behavior and feed efficiency traits.

Among a total of 26,076 quantitative trait loci (QTL) associated with 647 different traits reported in pigs (PigQTLdb^1^), 639 QTLs have been identified for feeding behavior and feed efficiency traits. Most of these QTL were identified using linkage mapping. Because of the large intervals of QTLs, directly using them for genetic improvement remains difficult (Tabor et al., 2002). In recent years, with the advent of dense marker panels, association mapping has become a powerful strategy for the detection of genetic variants associated with complex traits. It has been widely used in humans (McCarthy et al., 2008) and domestic animals (Andersson, 2009; Ma et al., 2014; Guo et al., 2016).

Duroc pig population is widely used as the terminal male parent of the DLY (Duroc × Landrace × Yorkshire) commercial pigs thanks to its excellent performance on growth traits. In previous studies, several single nucleotide polymorphisms (SNPs) that were significantly associated with FCR were detected on SSC 12 in a Canadian Duroc population by genome-wide association study (GWAS) (Ding et al., 2017). However, due to the limited sample size (<400), we detected only a few significant SNPs for feeding behavior and feed efficiency traits. In particular, the cost to measure feed efficiency traits has historically been the primary limitation to population-wide selection to improve feed efficiency in pigs (Bai et al., 2017). Here, we perform a GWAS in a larger American Duroc population to identify genetic variants associated with feeding behavior and feed efficiency traits.

## Materials and Methods

### Ethics Statement

The experimental procedures used in this study met the guidelines of the Animal Care and Use Committee of the South China Agricultural University (SCAU) (Guangzhou, China). The Animal Care and Use Committee of the SCAU approved all the animal experiments described in this study.

### Animals and Phenotype

During the period of 2013–2016, phenotypic data were collected for Duroc pigs (*n* = 1,008) from the Guangdong Wen’s Foodstuffs Group, Co., Ltd. (Guangdong, China) using the Osborne FIRE Pig Performance Testing System (Osborne, KS, United States) as previously described (Ding et al., 2017). Briefly, pigs were subjected to uniform feeding conditions for measurement of traits during the fattening period (approximately 11 weeks) from 30 to 100 kg live weight. Each animal was labeled a unique electric identification tag on its ear that could be captured by the automatic feeder. The time, duration, feed consumption, and BW of each individual were recorded at every visit to the feeder. Back fat (BF) of each animal was evaluated with a PIGLOG 105B ultrasound machine (SFK Technology, Søborg, Denmark) at the end of the test. The following feeding behavior and feed efficiency traits were defined and recorded for each pig: average DFI (kg/d), total daily time spent in feeder (TPD, min), number of daily visits to feeder (NVD), and FCR (Do et al., 2013a,b, 2014; Ding et al., 2017). RFI was computed using methods similar to those used by Cai et al. (2008). In the model, predicted DFI was estimated using linear regression of DFI on metabolic BW at mid-test (MWT), average daily gain from 30 to 100 kg (ADG), and BF. MWT was equal to [(BW at on-test + BW at off-test)/2]^0.75^. In summary, 1,008 pigs had phenotypic data for feeding behavior traits (DFI, TPD, and NVD), 981 for FCR and 971 for RFI.

### Genotyping and Quality Control

Genotyping was performed as described by Ding et al. (2017). Briefly, genomic DNA was extracted from ear tissue samples using the phenol–chloroform method. Genotyping was performed using the Geneseek Porcine 50K SNP Chip (Neogen, Lincoln, NE, United States), which contains 50,703 SNPs across autosomes and sex chromosomes. Quality control of the SNP data was performed using PLINK software (Purcell et al., 2007). Animals with call rates > 0.95, and SNPs with call rates > 0.99, minor allele frequency > 0.01, and *P*-value > 10^-6^ for the Hardy–Weinberg equilibrium test were included. Only autosomal SNPs were considered for subsequent analyses.

### GWAS and Genetic Analyses

GEMMA was used to perform GWAS with a univariate linear mixed model (Zhou and Stephens, 2012, 2014). Prior to GWAS, GEMMA was used to estimate the n × n standardized relatedness matrix (K) between the individuals. The following statistical model in matrix form was used: y = Wα + xβ + u + 𝜀, where *y* is the vector of phenotypic values for all pigs; *W* is the incidence matrices of covariates (fixed effects) including sex, pig pen, and year-season effects; α is the vector of corresponding coefficients including the intercept; *x* is the vector of marker genotypes, and β is the corresponding effect size of the marker; *u* is the vector of random effects, with *u* ∼ MVN_n_(0, λ τ^-1^K); 𝜀 is the vector of random residuals with 𝜀 ∼ MVN_n_(0, τ^-1^*I*_n_); τ^-1^ is the variance of the residual errors; λ is the ratio between the two variance components; *K* is a known n × n relatedness matrix; and *I* is an n × n identity matrix. MVN_n_ denotes the n-dimensional multivariate normal distribution.

Genome-wide significance was determined using the Bonferroni method by dividing the desired type I error level by the number of SNPs tested. To include additional candidate genes and enable gene set enrichment analysis, we also set a more lenient threshold by multiplying the Bonferroni threshold by a constant of 20 (Yang et al., 2005).

The GCTA tool was used to compute the genomic heritability by dividing the estimated genetic variance by the total variance measured, and phenotypic variances contributed by significant SNPs for each trait (Yang et al., 2010, 2011). Genetic correlation was estimated using GCTA in the bivariate mode.

A number of SNPs that were significantly associated with the target trait by GWAS were detected based on their strong linkage with highly causal mutants. To demarcate the independence of all significant signals in a putative region, conditional and LD analyses were performed in univariate linear mixed models by fitting the genotypes of peak SNPs as covariates (Yang et al., 2012). Moreover, to further detect candidate regions associated with feeding behavior and feed efficiency traits, PLINK (Purcell et al., 2007) and Haploview (Barrett et al., 2005) were utilized for haplotype block analysis. LD blocks were defined using the solid spin algorithm by the criteria of Gabriel et al. (2002).

### Annotation of SNPs

All SNP location on the *Sus scrofa* 10.2 genome version were downloaded from Ensembl. The Ensembl annotation of the *S. scrofa* 10.2 genome version was employed to find genes that were nearest to the significant SNPs^1^. To annotate significant SNP located in previously mapped QTLs in pigs, all QTL data in pigs were downloaded from http://www.animalgenome.org/cgi-bin/QTLdb/SS/download?file=gbpSS_10.2 (accessed on December 18, 2017) (Hu et al., 2013). For functional annotation, Kyoto Encyclopedia of Genes and Genomes (KEGG) and Gene Ontology analyses were used for the identification of related pathways. KEGG and GO analyses were performed on KOBAS 3.0 (Xie et al., 2011). Fisher’s exact test was used to assess the significance of the enriched terms, and *P* < 0.05 was selected to explore the genes involved in biological processes (Xing et al., 2016; Wang et al., 2017).

## Results

### Quantitative Genetics of Feeding Behavior and Feed Efficiency Traits in Pigs

We considered five feeding behavior and feed efficiency traits, including DFI, TPD, NVD, FCR, and RFI. Summary statistics for these traits, and their heritabilities are presented in **Table 1**. All phenotypic data conformed to the Gaussian distribution based on the Shapiro test before GWAS analysis (Theune, 1973). There existed substantial phenotypic variation, with coefficient of variation (CV) ranging from 10 to 27% for the five traits (**Table 1**).

**Table 1:** Summary statistics of feeding behavior and feed efficiency in a Duroc population.

Trait^1^	Unit	*N*	Mean ±*SD*	Max	Min	C.V.
DFI	kg	1008	1.96 ± 0.19	3.11	1.13	9.69
TPD	Min	1008	60.58 ± 9.71	104.48	32.5	16.03
NVD	count	1008	6.92 ± 1.86	16.53	3.27	26.88
FCR	kg/kg	981	2.25 ± 0.22	2.95	1.62	9.78
RFI	kg	971	0 ± 0.14	0.42	-0.93	–

*^*1*^DFI, total daily feed intake; TPD, total time spent at feeder per day; NVD, number of visits to the feeder per day; FCR, feed conversion ratio; RFI, residual feed intake. Mean (standard deviation), maximum (max), minimum (min), and coefficient of variation (C.V.) values are presented for all of the phenotypes included in the association study (N).*

We partitioned the phenotypic variance into genetic and environmental components using genetic relationship matrix computed from genotypes. The genomic heritabilities of the traits are moderate, ranging from 0.28 (DFI) to 0.38 (NVD) (**Table 2**). Bivariate analysis indicated that the traits are positively correlated with each other, both phenotypically and genetically, except between DFI and NVD. In particular, DFI are strongly positively correlated with FCR and RFI with a genetic correlation of 0.75 and 0.98, respectively (**Table 2**).

**Table 2:** Heritability, genetic and phenotypic correlations for feeding behavior and feed efficiency traits.

Trait	DFI	TPD	NVD	FCR	RFI
DFI	**0.28 ± 0.06**	0.31	-0.4	0.34	0.81
TPD	0.67 ± 0.07	**0.37 ± 0.06**	0.15	0.11	0.30
NVD	-0.08 ± 0.11	0.38 ± 0.09	**0.38 ± 0.05**	0.12	0.04
FCR	0.75 ± 0.06	0.22 ± 0.11	0.33 ± 0.10	**0.32 ± 0.06**	0.73
RFI	0.98 ± 0.01	0.63 ± 0.07	0.14 ± 0.11	0.97 ± 0.01	**0.31 ± 0.03**

*Corresponding SD behind the mean heritabilities on diagonal (bold), phenotypic correlations above diagonal, genetic correlations below diagonal.*

### Genome-Wide Association Studies

To understand the genetic architecture of the feeding behavior and feed efficiency traits, we performed GWAS for each trait. After a series of filtering steps for quality control, 32,446 SNPs and 1,008 pigs were available for subsequent GWAS. The number of SNPs on each chromosome and the average distances between pairs of SNPs after QC are provided in **Supplementary Table S1**. The average physical distance between two neighboring SNPs on the same chromosome was approximately 75.3 kb and ranged from 64.5 (SSC11) to 112.3 kb (SSC1).

**FIGURE 1:**
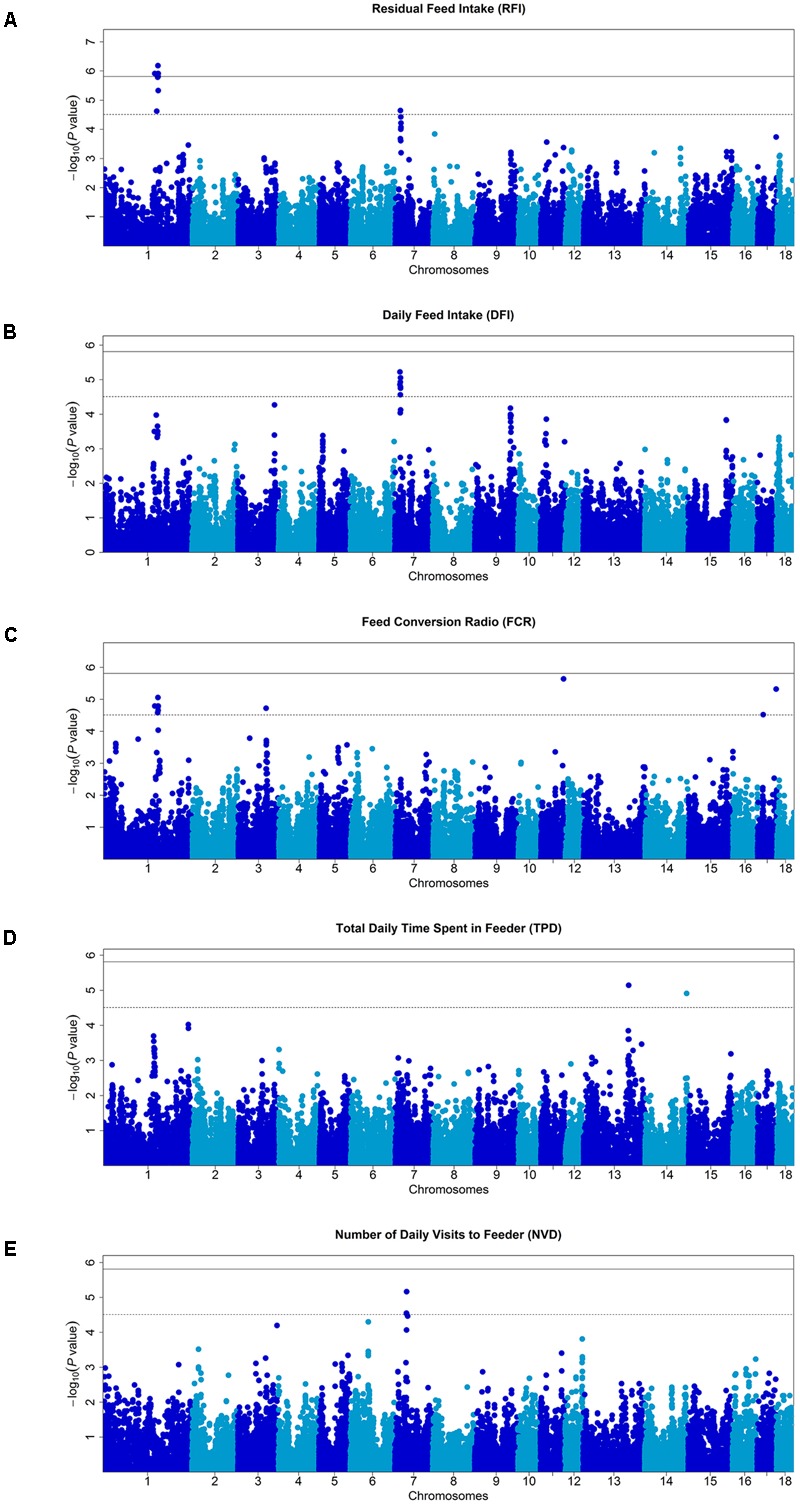
Manhattan plots of genome-wide association studies for feeding behavior and feed efficiency in male Duroc pigs. In the Manhattan plots, negative log10 *P*-values of the quantified SNPs were plotted against their genomic positions. Different colors indicate various chromosomes. The solid and dashed lines indicate the 5% genome-wide and chromosome-wide Bonferroni-corrected thresholds, respectively. On the vertical axis, Manhattan plot for **(A)** residual feed intake (RFI), **(B)** total daily feed intake (DFI), **(C)** feed conversion ratio (FCR), **(D)** total daily time spent in feeder (TBD), and **(E)** number of visits to feeder (NVD), respectively.

Single marker tests using mixed model were performed to identify genetic markers associated with the traits. At a stringent genome-wide Bonferroni threshold *P* < 1.54E-06 (0.05/32,446), 9 SNPs on SSC1 were associated with RFI. At a more lenient threshold (*P* < 3.08E-05) for suggestive associations, 8 SNPs were associated with DFI, 15 with FCR, 5 with NVD, 2 with TPD, and 14 with RFI (**Figure 1** and **Table 2**). QQ plots of *P*-values and the computed genomic inflation factors (λ) indicated no evidence of population stratification (Pearson and Manolio, 2008; Utsunomiya et al., 2013) (**Supplementary Figure S1**).

**FIGURE 2:**
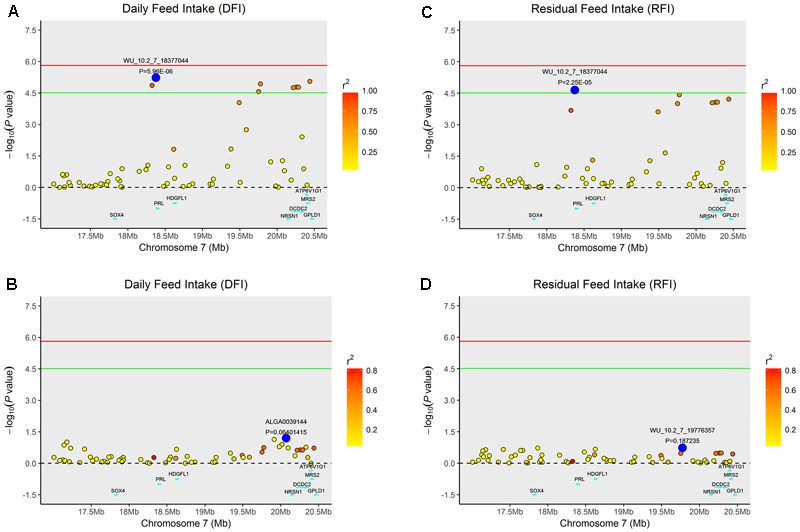
Regional association plot of the primary signal (WU_10.2_7_18377044) associated with DFI and RFI at SSC7. For each plot, negative log10 *P*-values of SNPs (y-axis) are presented according to their chromosomal positions (x-axis). The red line and green line indicate the genome-wide significance level (*P* < 1.54E-06) and the suggestive level (*P* < 3.08E-05), respectively. The primary SNP are denoted by large blue circles. SNPs are represented by colored circles according to the target SNP with which they were in strongest LD. The left panel of the figure shows the association results for DFI **(A)** before and **(B)** after conditional analysis on WU_10.2_7_18377044. The right panel of the figure shows the association results for RFI **(C)** before and **(D)** after conditional analysis on WU_10.2_7_18377044. The *P*-value of association results for **(B)** DFI and **(D)** RFI after conditional analysis on WU_10.2_7_18377044 fell below the predicted threshold.

Multiple SNPs in close proximity were found to be associated with the same traits, possibly due to their linkage and/or linkage disequilibrium. Indeed, LD block analysis showed that the multiple significant SNPs on SSC1 associated with RFI were located within a haplotype block that spanned 2,169 kb (**Figure 4**). To test whether LD caused the associations, we performed conditional analyses with DFI and RFI in which the lead SNP WU_10.2_7_18377044 on SSC7 was fitted in the model as a covariate and the conditional *P*-values for other SNPs in the vicinity were obtained. While many SNPs in high LD with the lead SNP were significant in the GWAS for DFI (**Figure 2A**), their significance almost diminished completely after the lead SNP was included as a fixed effect in the model (**Figure 2B**). The same pattern was also observed for the same lead SNP for RFI (**Figures 2C,D**). This is not surprising because the two DFI and RFI were almost perfectly (*r* = 0.98) correlated genetically (**Table 2**).

**FIGURE 3:**
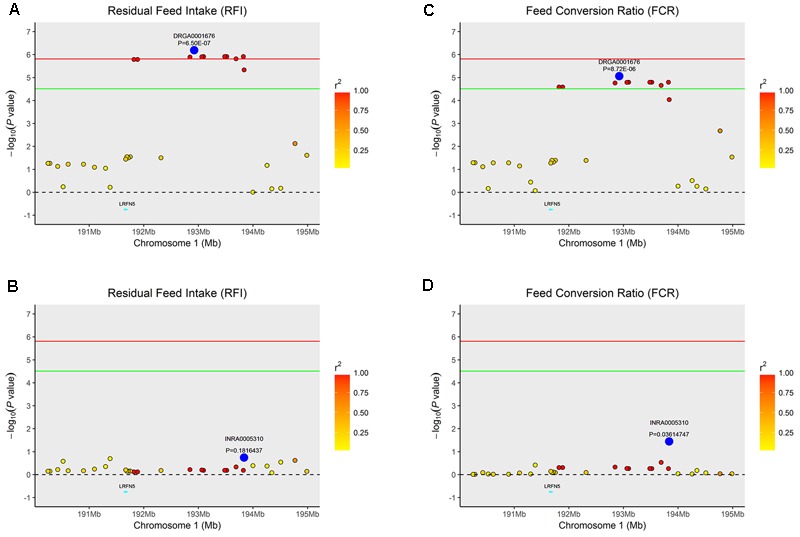
Regional association plot of the primary signal (DRGA0001676) associated with FCR and RFI at SSC1. For each plot, negative log10 *P*-values of SNPs (y-axis) are presented according to their chromosomal positions (x-axis). The red line and green line indicate the genome-wide significance level (*P* < 1.54E-06) and the suggestive level (*P* < 3.08E-05), respectively. The primary SNP are denoted by large blue circles. SNPs are represented by colored circles according to the target SNP with which they were in strongest LD. The left panel of the figure shows the association results for FCR **(A)** before and **(B)** after conditional analysis on DRGA0001676. The right panel of the figure shows the association results for RFI **(C)** before and **(D)** after conditional analysis on DRGA0001676. The *P*-value of association results for **(B)** FCR and **(D)** RFI after conditional analysis on DRGA0001676 fell below the predicted threshold.

Similarly, the lead SNP DRGA0001676 significantly associated with RFI and FCR in SSC1 explained the association between multiple SNPs with the traits in the vicinity of DRGA0001676 (**Figure 3**).

**FIGURE 4:**
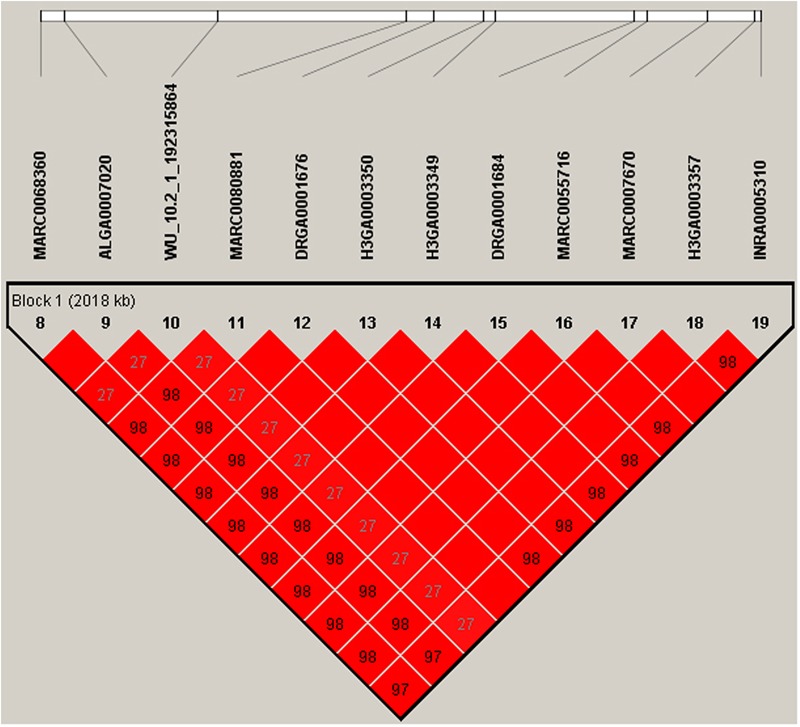
The LD block in the significantly associated region on SSC1. LD blocks are marked with triangles. Values in boxes are LD (r^2^) between SNP pairs and the boxes are colored according to the standard Haploview color scheme: LOD > 2 and D′ = 1, red; LOD > 2 and D′ < 1, shades of pink/red; LOD < 2 and D′ = 1, blue; LOD < 2 and D′ < 1, white (LOD is the log of the likelihood odds ratio, a measure of confidence in the value of D′). Annotated genes in the chromosomal region retrieved from the Ensemble genome browser (www.ensembl.org/Sus_scrofa/Info/Index).

### Comparison With Previously Mapped Pig QTLs

To evaluate whether SNPs associated with the feeding behavior and feed efficiency trait in this study replicate any previously known QTLs, we search the pigQTLdb based on SNP and QTL locations. A total of 13 SNPs associated with FCR and/or RFI were identified within the genomic regions where QTLs for DFI have been previously mapped in pigs (**Table 3**). Eight SNPs associated with DFI and RFI were located on previously reported QTL regions for time spent drinking. One SNP on SSC13 associated with TPD was located on previously reported QTL regions for FCR in pigs. Moreover, 10 SNPs were located in the genomic regions where QTLs were previously detected by GWAS or linkage mapping for average daily gain and growth-related traits in pigs. Given the high genetic correlations (**Table 2**), overlaps were considered with QTLs for correlated traits as evidence for replication.

**Table 3:** Tag SNPs and Candidate genes for feeding behavior and feed efficiency traits.

Trait	SNP ID	SSC^1^	Location (bp)^2^	Explained genetic variance (%)	*P*-value^3^	Distance/bp^4^	Candidate gene
DFI	WU_10.2_7_18377044	7	18,377,044	2.16	5.96E-06	35449	*PRL*
	DRGA0007294	7	20,439,045	1.85	8.79E-06	Within	*MRS2*
	WU_10.2_7_19776357	7	19,776,357	1.75	1.16E-05	380549	*NRSN1*
	WU_10.2_7_18325943	7	18,325,943	2.12	1.37E-05	86550	*PRL*
	H3GA0020180	7	20,261,277	1.70	1.62E-05	Within	*DCDC2*
	ASGA0031614	7	20,287,356	1.69	1.67E-05	Within	*DCDC2*
	ASGA0031606	7	20,216,211	1.69	1.76E-05	Within	*DCDC2*
	WU_10.2_7_19752202	7	19,752,202	1.69	2.71E-05	404,704	*NRSN1*
FCR	DRGA0011514	11	80,745,166	2.97	2.28E-06	819,916	*EFNB2*
	WU_10.2_17_66292358	17	66,292,358	3.59	4.75E-06	11,651	*GNAS*
	DRGA0001676	1	192,923,645	3.22	8.72E-06	-1,220,058	*LRFN5*
	ALGA0007029	1	181,869,453	3.11	1.61E-05	-169,542	*MEGF11*
	H3GA0003349	1	193,093,994	3.11	1.61E-05	-1,390,407	*LRFN5*
	DRGA0001684	1	193,482,814	3.11	1.61E-05	1,044,746	*FSCB*
	MARC0055716	1	193,518,898	3.11	1.61E-05	1,008,662	*FSCB*
	H3GA0003357	1	193,823,157	3.11	1.61E-05	704,403	*FSCB*
	H3GA0003350	1	193,062,563	3.11	1.63E-05	-1,358,976	*LRFN5*
	MARC0080881	1	192,844,903	3.09	1.74E-05	-1,141,316	*LRFN5*
	ASGA0095444	3	98,844,612	2.85	1.89E-05	-112,210	*MSH6*
	MARC0007670	1	193,689,396	3.09	2.21E-05	838,164	*FSCB*
	MARC0068360	1	191,819,089	2.97	2.60E-05	-115,502	*LRFN5*
	ALGA0007020	1	191,885,797	2.97	2.60E-05	-182,210	*LRFN5*
	ALGA0093563	17	20,141,183	2.66	2.99E-05	-280,271	*PLCB1*
NVD	MARC0042115	7	41,854,212	1.50	6.78E-06	-8422	*NCR2*
	WU_10.2_7_41505385	7	41,505,385	1.52	2.80E-05	-18,306	*APOBEC2*
	H3GA0021155	7	41,720,015	1.52	2.89E-05	Within	*ENSSSCG00000001612*
	WU_10.2_7_41803542	7	41,803,542	1.55	2.89E-05	Within	*TREM1*
	H3GA0021194	7	42,150,905	1.50	2.93E-05	-8973	*MDFI*
TPD	ASGA0059147	13	159,897,035	3.78	7.16E-06	30,566	*DZIP3*
	WU_10.2_14_147097059	14	147,097,059	1.30	1.22E-05	-51,628	*ADAM12*
RFI	DRGA0001676	1	192,923,645	5.46	6.50E-07	-1,220,058	*LRFN5*
	ALGA0007029	1	181,869,453	5.37	1.21E-06	-169,542	*MEGF11*
	H3GA0003349	1	193,093,994	5.37	1.21E-06	-1,390,407	*LRFN5*
	DRGA0001684	1	193,482,814	5.37	1.21E-06	1,044,746	*FSCB*
	MARC0055716	1	193,518,898	5.37	1.21E-06	1,008,662	*FSCB*
	H3GA0003357	1	193,823,157	5.37	1.21E-06	704,403	*FSCB*
	H3GA0003350	1	193,062,563	5.36	1.25E-06	-1,358,976	*LRFN5*
	MARC0080881	1	192,844,903	5.36	1.28E-06	-1,141,316	*LRFN5*
	MARC0007670	1	193,689,396	5.43	1.53E-06	838,164	*FSCB*
	MARC0068360	1	191,819,089	5.22	1.63E-06	-115,502	*LRFN5*
	ALGA0007020	1	191,885,797	5.22	1.63E-06	-182,210	*LRFN5*
	INRA0005310	1	193,837,874	5.01	4.67E-06	689,686	*FSCB*
	WU_10.2_7_18377044	7	18,377,044	2.37	2.25E-05	35,449	*PRL*
	WU_10.2_1_188782437	1	188782437	4.37	2.37E-05	-154923	FBXO33

*^*1*^*Sus scrofa* chromosome. ^*2*^SNP positions in Ensembl. ^*3*^Genome-wide significant associations are underlined. ^*4*^+/-: The SNP located upstream/downstream of the nearest gene; NA, not assigned.*

### Candidate Genes and Functional Analysis

A total of 16 functional genes that were within or near the identified tag SNPs were detected based on annotations of the *Sus scrofa* 10.2 genome assembly (**Table 2**). Many candidate genes appear to have biochemical and physiological roles that were are relevant to feeding behavior and feed efficiency traits, including neurensin 1 (*NRSN1*) and doublecortin domain-containing 2 (*DCDC2*) for DFI; ADAM metallopeptidase domain 12 (*ADAM12*) for TPD; phospholipase C beta 1 (*PLCB1*), GNAS complex locus (*GNAS*), and ephrin B2 (*EFNB2*) for FCR; prolactin (*PRL*) for both FCR and RFI; leucine-rich repeat and fibronectin type III domain-containing 5 (*LRFN5*), and multiple EGF-like domains 11 (*MEGF11*) for RFI and FCR.

To uncover genes and pathways involved in the biology of feed efficiency and feeding behavior traits, we performed gene set enrichment analysis of the 16 candidate genes for all five traits. Interestingly, at an FDR = 0.05, several KEGG pathways and GO terms are significantly enriched for the candidate genes, including pathways related to hormone metabolic process, secretion of digestive enzymes, among others (**Supplementary Table S2**).

## Discussion

In this study, we performed a GWAS for five feeding behavior and feed efficiency traits. We identified a number of genetic markers and genes associated with the traits. Gene set enrichment analysis revealed pathways that appear to be consistent with the underlying biology of the traits. This represents a first step toward understanding the genetic basis of feeding behaviors and feed efficiency and developing informative genetic markers for efficient breeding programs.

Despite similar design and analytical methods, none of the QTLs identified in this study replicated QTLs in a previous study (Ding et al., 2017). There could be a number of reasons. First, this study has a substantially larger sample size (1,008 vs. 338). Second, while both studies used Duroc pigs, the genetic backgrounds have subtle differences. For example, Duroc boars in the previous study (Ding et al., 2017) was of Canadian origin while pigs in the present study were of American origin. It is well-established that genetic backgrounds can have substantial influence on single marker associations. Moreover, this study detected QTLs that overlapped previously identified QTLs (**Table 4**). Indeed, over 87% of SNPs (28/32) significantly associated with feeding behavior and feed efficiency traits were located within genomic regions where QTLs for feeding behavior and feed efficiency were previously reported in pigs (pigQTLdb^2^).

**Table 4:** Comparative mapping of tag SNPs with previous QTLs reported in the pig QTL database (as of December 18, 2017) and previous GWAS results.

SSC^1^	SNP ID	Location (bp)^2^	QTL location range^3^	QTL ID^4^	Related QTL	Trait
1	DRGA0001676	192923645	1: 162073157–233806417	141	Daily feed intake	RFI/FCR
1	ALGA0007029	181869453	1: 162073157–233806417	141	Daily feed intake	RFI/FCR
1	H3GA0003349	193093994	1: 162073157–233806417	141	Daily feed intake	RFI/FCR
1	DRGA0001684	193482814	1: 162073157–233806417	141	Daily feed intake	RFI/FCR
1	MARC0055716	193518898	1: 162073157–233806417	141	Daily feed intake	RFI/FCR
1	H3GA0003357	193823157	1: 162073157–233806417	141	Daily feed intake	RFI/FCR
1	H3GA0003350	193062563	1: 162073157–233806417	141	Daily feed intake	RFI/FCR
1	MARC0080881	192844903	1: 162073157–233806417	141	Daily feed intake	RFI/FCR
1	MARC0007670	193689396	1: 162073157–233806417	141	Daily feed intake	RFI/FCR
1	MARC0068360	191819089	1: 162073157–233806417	141	Daily feed intake	RFI/FCR
1	ALGA0007020	191885797	1: 162073157–233806417	141	Daily feed intake	RFI/FCR
1	INRA0005310	193837874	1: 162073157–233806417	141	Daily feed intake	RFI
1	WU_10.2_1_188782437	188782437	1: 162073157–233806417	141	Daily feed intake	RFI
7	WU_10.2_7_18377044	18377044	7: 11625414–36993248	5916	Time spent drinking	DFI/RFI
7	DRGA0007294	20439045	7: 11625414–36993248	5916	Time spent drinking	DFI
7	WU_10.2_7_19776357	19776357	7: 11625414–36993248	5916	Time spent drinking	DFI
7	WU_10.2_7_18325943	18325943	7: 11625414–36993248	5916	Time spent drinking	DFI
7	H3GA0020180	20261277	7: 11625414–36993248	5916	Time spent drinking	DFI
7	ASGA0031614	20287356	7: 11625414–36993248	5916	Time spent drinking	DFI
7	ASGA0031606	20216211	7: 11625414–36993248	5916	Time spent drinking	DFI
7	WU_10.2_7_19752202	19752202	7: 11625414–36993248	5916	Time spent drinking	DFI
7	MARC0042115	41854212	7: 38992356–48448805	191	Average daily gain	NVD
7	WU_10.2_7_41505385	41505385	7: 38992356–48448805	191	Average daily gain	NVD
7	H3GA0021155	41720015	7: 38992356–48448805	191	Average daily gain	NVD
7	WU_10.2_7_41803542	41803542	7: 38992356–48448805	191	Average daily gain	NVD
7	H3GA0021194	42150905	7: 38992356–48448805	191	Average daily gain	NVD
13	ASGA0059147	159897035	13: 130538722–194995520	2884	Feed conversion ratio	TPD
17	WU_10.2_17_66292358	66292358	17: 64337706–66928144	3829	Average daily gain	FCR

*^*1*^*Sus scrofa* chromosome. ^*2*^SNP positions in Ensembl. ^*3*^Location range of the mapped QTL in the QTL database. ^*4*^Identity of QTL in the pig QTL database or published literature.*

Derivative traits are correlated with each other. For example, RFI and DFI has a highly correlated at 0.81 phenotypically and 0.98 genetically. Indeed, GWAS signals overlapped to some extent for these two traits (**Figures 1A,B**). Signals are detected for both traits on SSC7. And while the strengths of associations differ and may not reach thresholds for both traits, SNPs associated with RFI on SSC1 also tended to have an effect for DFI (**Figures 1A,B**). However, because of the less than unity correlations, in particular the correlation at the phenotypic level which were used for GWAS mapping, full overlap is not expected.

Because of the low to moderate heritabilities of these traits, informative markers are particularly useful in selection programs. Our analyses indicated that the markers WU_10.2_7_18377044 and DRGA0001676 were two important polymorphisms with pleiotropic effects. SNP WU_10.2_7_18377044 on SSC7 explained 2.16 and 2.37% of the observed phenotypic variance for DFI and RFI, respectively. Another SNP DRGA0001676 on SSC1 explained 3.22 and 5.46% of the observed phenotypic variance for FCR and RFI, respectively. Although causal variants remain to be identified, given the substantial phenotypic variance explained, these SNPs could potentially serve as genetic markers in breeding programs.

Feeding is one of the most conserved activities of animals, and regulating feed intake is a fundamental process for animal survival (Bader et al., 2007). Physiological modulation is often accomplished through the regulation of the nervous system (Destexhe and Marder, 2004). In mammals, the hypothalamus controls appetite and satiety by integrating neuropeptides or hormonal signals (Berthoud, 2002; Leibowitz and Wortley, 2004; Fang et al., 2011). Our GWAS implicated several genes that have roles in neural development. The gene *NRSN1* associated with DFI plays an important role in neural organelle transport and in the transduction of nerve signals or in nerve growth (Yu and Kaang, 2016; Lencer et al., 2017). Three significant SNPs located within the *DCDC2* gene are associated with DFI. *DCDC2* is involved in the conduction of neural signals and the development of neurons (Meng et al., 2005). Takasu et al. (2002) reported that EFNB2 protein, which is associated with FCR, is localized at excitatory synapses, regulating the development and remodeling of neural signals. *MEGF11*, another gene associated with FCR, is highly expressed in the central nervous system, whose homology *MEGF10* in Drosophila plays an important role in maintaining the normal functioning of the brain (Draper et al., 2014). Finally, *LRFN5*, also a FCR associated gene, is a key neurodevelopmental gene that is associated with developmental delay (Mikhail et al., 2011).

Daily feed intake is a major concern of breeders because it is highly correlated with growth rate and feed efficiency (Do et al., 2013a). The most significant locus associated with DFI and RFI on SSC 7, WU_10.2_7_18377044, is close to the *PRL* gene, which encodes the anterior pituitary hormone prolactin. The secreted hormone is a growth regulator for many tissues (McAtee and Trenkle, 1971; Kennaway et al., 1982). Yayou et al. (2011) reported that administration of prolactin-releasing peptide in the central nervous system will result in reduced feed intake and abnormal feeding behavior in steers. In goats, *prolactin* has also been found to affect feed intake (Sergent et al., 1988).

Growth rate and feed intake were major influencing factors of FCR. Among the candidate genes identified by GWAS, *PLCB1* is a protein-coding gene near the SNP ALGA0093563. Meng et al. (2017) reported that this gene is significantly associated with ADG in a Yorkshire pig and may be involved in pig growth and development. The *GNAS* gene is near SNP WU_10.2_17_66292358. The imprinted *GNAS* is involved in obesity, energy metabolism, feeding behavior, and viability. Eaton et al. (2012) reported that mutations in this gene can lead to pre-weaning growth retardation and incomplete catch-up growth in mice.

Feeding time is an important feeding behavior trait that directly affects the growth rate and efficiency. ADAM metallopeptidase domain 12 (*ADAM12*) is located proximal to the SNP WU_10.2_14_14709705, which is associated with TPD. *ADAM12* had been identified as a protease to insulin-like growth factor (IGF)-binding proteins. This gene may have a regulatory function in controlling the amount of free bioactive IGF (Cowans and Spencer, 2007). In chickens, insulin has been consistently shown to affect feed intake and feed behavior (Shiraishi et al., 2008, 2011).

Functional annotation revealed a number of pathways and biological processes that are significantly overrepresented among the 16 positional candidate genes for feeding behavior and feed efficiency traits (**Supplementary Table S2**). Most of the significantly enriched pathways are associated with hormonal and digestive gland secretion during feeding such as thyroid hormone and gastric acid secretion. Given the principal roles of hormone and digestive gland secretion in feeding and metabolism, their involvement in the feeding behavior and feed efficiency traits are conceivable.

Several GWAS for feeding behavior and feed efficiency traits in Duroc populations with different genetic backgrounds have been reported (Do et al., 2013a,b; Ding et al., 2017; Quan et al., 2017). We previously found, by GO enrichment analysis, that most of the potential candidate genes were involved in the development of the hypothalamus (Ding et al., 2017). This agrees with our findings that feeding behavior is mainly controlled by the central nervous system. Results in the present study further extend to suggest that the hormonal and digestive glands are also involved. However, functional studies remain to be performed to delineate the mechanisms of how the constellations of genes implicated by the GWAS affect feeding.

## Author Contributions

ZW, JY, and WH: conceived and designed the experiments. RD, MY, XW, JQ, ZZ, SZ, and SL: performed the experiments. RD, JY, and WH: analyzed the data and wrote manuscript. MY, ZX, EZ, GC, and DL: collected the samples and recorded the phenotypes. ZW: contributed materials. All authors reviewed and approved the manuscript.

## Conflict of Interest Statement

The authors have read the journal’s policy and declare the following conflicts of interest: Wens Foodstuffs Group, Co., Ltd. provided all phenotypic data and ear tissue samples for the research reported in this study. This does not alter our adherence to Frontiers in Genetics policies on sharing data and materials.

## Footnotes

1 http://ensembl.org/Sus_scrofa/Info/Index

2 https://www.animalgenome.org/cgi-bin/QTLdb/SS/index

## Supplementary Material

FIGURE S1 Quantile–quantile (Q–Q) plots of genome-wide association studies for feeding behavior and feed efficiency in male Duroc pigs. Q–Q plots show the observed versus expected negative log10 *P*-values. On the vertical axis, Q–Q plot for **(A)** residual feed intake (RFI), **(B)** total daily feed intake (DFI), **(C)** feed conversion ratio (FCR), **(D)** total daily time spent in feeder (TBD), and **(E)** number of visits to feeder (NVD), respectively.

TABLE S1 Distributions of SNPs after QC and the average distance between adjacent SNPs on each chromosome.

TABLE S2 Significant KEGG PATHWAY and GO terms with feeding behavior and feed efficiency traits (*P*-value < 0.05).
