# Supplementary figures and images for: Genetic Architecture of Feeding Behavior and Feed Efficiency in a Duroc Pig Population
Source: Front Genet. 2018 Jun 19;9:220. doi: 10.3389/fgene.2018.00220 (PMC6018414; doi:10.3389/fgene.2018.00220)

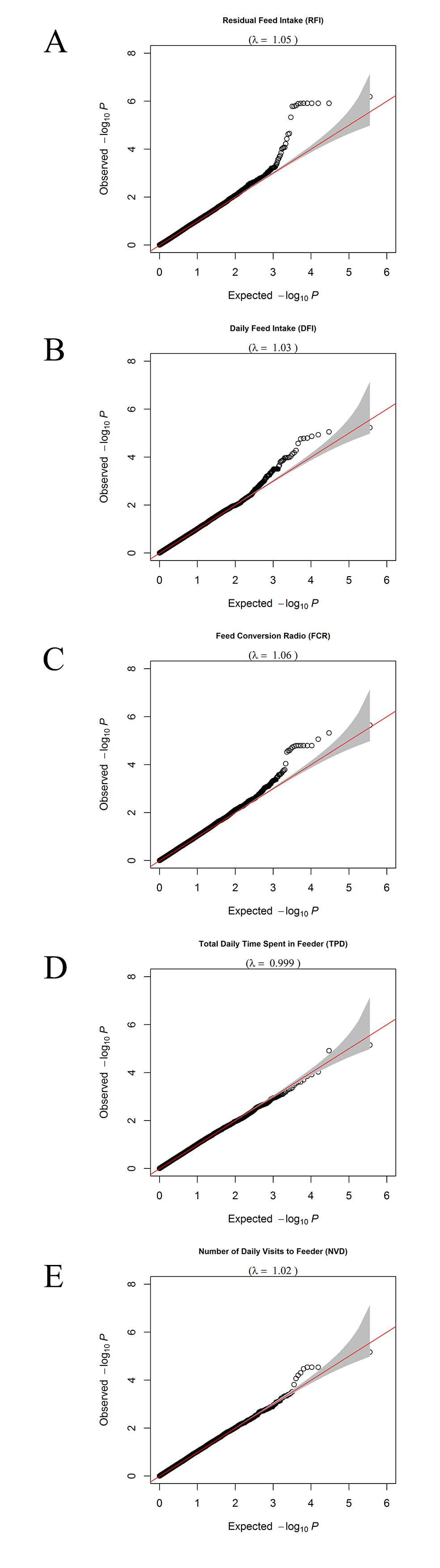

Supplement: FIGURE S1 — Quantile–quantile (Q–Q) plots of genome-wide association studies for feeding behavior and feed efficiency in male Duroc pigs. Q–Q plots show the observed versus expected negative log10 P-values. On the vertical axis, Q–Q plot for (A) residual feed intake (RFI), (B) total daily feed intake (DFI), (C) feed conversion ratio (FCR), (D) total daily time spent in feeder (TBD), and (E) number of visits to feeder (NVD), respectively. [file Image_1.JPEG]
