# Supplementary material for: Genetic Architecture of Feeding Behavior and Feed Efficiency in a Duroc Pig Population
Source: Front Genet. 2018 Jun 19;9:220. doi: 10.3389/fgene.2018.00220 (PMC6018414; doi:10.3389/fgene.2018.00220)
Supplement: TABLE S1 — Distributions of SNPs after QC and the average distance between adjacent SNPs on each chromosome. [file Table_1.DOCX]

**Supplementary Materials**

**Genetic Architecture of Feeding Behavior and Feed Efficiency in a Duroc Pig Population**

Rongrong Ding^1,#^, Ming Yang^2,#^, Xingwang Wang^1^, Jianping Quan^1^, Zhanwei Zhuang^1^, Shenping Zhou^1^, Shaoyun Li^1^, Zheng Xu^1^, Enqin Zheng^1^, Gengyuan Cai^1,2^, Dewu Liu^1^, Wen Huang^3^, Jie Yang^1,*^, Zhenfang Wu^1,2,*^

^1^College of Animal Science and National Engineering Research Center for Breeding Swine Industry, South China Agricultural University, Guangdong 510642, P.R. China.

^2^National Engineering Research Center for Breeding Swine Industry, Guangdong Wens Foodstuffs Group Co., Ltd, Guangdong 527400, P.R. China.

^3^Department of Animal Science, Michigan State University, East Lansing, MI 48824, USA

^#^These authors contributed equally to this work.

**Correspondence and requests for materials should be addressed to J.Y. (email: jieyang2012@hotmail.com) or Z.W. (email: wzfemail@163.com)*

**S1 Table. Distributions of SNPs after QC and the average distance between adjacent SNPs on each chromosome.**

| **SSC** | **SNP no.** | **Physical size (Mb)^1^** | | **Mb/SNP** |
| --- | --- | --- | --- | --- |
| 1 | 2805 | 314.93 | 0.1123 | |
| 2 | 2212 | 162.28 | 0.0734 | |
| 3 | 1988 | 143.99 | 0.0724 | |
| 4 | 2077 | 143.40 | 0.0690 | |
| 5 | 1649 | 111.23 | 0.0675 | |
| 6 | 2259 | 157.53 | 0.0697 | |
| 7 | 2042 | 134.51 | 0.0659 | |
| 8 | 2044 | 147.27 | 0.0720 | |
| 9 | 2097 | 153.43 | 0.0732 | |
| 10 | 1198 | 77.73 | 0.0649 | |
| 11 | 1359 | 87.63 | 0.0645 | |
| 12 | 965 | 63.41 | 0.0657 | |
| 13 | 2321 | 218.12 | 0.0940 | |
| 14 | 2222 | 153.53 | 0.0691 | |
| 15 | 2030 | 157.40 | 0.0775 | |
| 16 | 1270 | 86.69 | 0.0683 | |
| 17 | 1003 | 69.32 | 0.0691 | |
| 18 | 905 | 60.91 | 0.0673 | |
| Total | 32446 | 2443.31 |  | |

SNP, single nucleotide polymorphisms; SSC, *Sus scrofa* chromosome

^1^The physical size is based on *Sus scrofa* Build 10.2 (http://www.ensembl.org/Sus_scrofa/Info/Index)

**S2 Table: Significant KEGG PATHWAY and GO terms with feeding behavior and feed efficiency traits. (*P*-value<0.05)**

| **Term** | **Database** | **ID** | **Gene names** | **Corrected P-Value** |
| --- | --- | --- | --- | --- |
| [Endocrine and other factor-regulated calcium reabsorption](http://www.genome.jp/kegg-bin/show_pathway?ssc04961/ssc:100152679%09red/ssc:100049657%09red) | KEGG PATHWAY | ssc04961 | *GNAS, PLCB1* | 0.006746 |
| [Long-term depression](http://www.genome.jp/kegg-bin/show_pathway?ssc04730/ssc:100152679%09red/ssc:100049657%09red) | KEGG PATHWAY | ssc04730 | *GNAS, PLCB1* | 0.006746 |
| [Renin secretion](http://www.genome.jp/kegg-bin/show_pathway?ssc04924/ssc:100152679%09red/ssc:100049657%09red) | KEGG PATHWAY | ssc04924 | *GNAS, PLCB1* | 0.006746 |
| [Salivary secretion](http://www.genome.jp/kegg-bin/show_pathway?ssc04970/ssc:100152679%09red/ssc:100049657%09red) | KEGG PATHWAY | ssc04970 | *GNAS, PLCB1* | 0.006746 |
| [Thyroid hormone synthesis](http://www.genome.jp/kegg-bin/show_pathway?ssc04918/ssc:100152679%09red/ssc:100049657%09red) | KEGG PATHWAY | ssc04918 | *GNAS, PLCB1* | 0.006746 |
| [Gastric acid secretion](http://www.genome.jp/kegg-bin/show_pathway?ssc04971/ssc:100152679%09red/ssc:100049657%09red) | KEGG PATHWAY | ssc04971 | *GNAS, PLCB1* | 0.006746 |
| [Insulin secretion](http://www.genome.jp/kegg-bin/show_pathway?ssc04911/ssc:100152679%09red/ssc:100049657%09red) | KEGG PATHWAY | ssc04911 | *GNAS, PLCB1* | 0.006746 |
| [Pancreatic secretion](http://www.genome.jp/kegg-bin/show_pathway?ssc04972/ssc:100152679%09red/ssc:100049657%09red) | KEGG PATHWAY | ssc04972 | *GNAS, PLCB1* | 0.006746 |
| [Glucagon signaling pathway](http://www.genome.jp/kegg-bin/show_pathway?ssc04922/ssc:100152679%09red/ssc:100049657%09red) | KEGG PATHWAY | ssc04922 | *GNAS, PLCB1* | 0.006746 |
| [Response to parathyroid hormone](http://amigo.geneontology.org/cgi-bin/amigo/term_details?term=GO:0071107) | Gene Ontology | GO:0071107 | *GNAS* | 0.003388 |
| [Regulation of hormone metabolic process](http://amigo.geneontology.org/cgi-bin/amigo/term_details?term=GO:0032350) | Gene Ontology | GO:0032350 | *PRL* | 0.016839 |
| Negative regulation of hormone metabolic process | Gene Ontology | GO:0032351 | *PRL* | 0.006766 |
| Negative regulation of hormone biosynthetic process | Gene Ontology | GO:0032353 | *PRL* | 0.006766 |
| Regulation of hormone biosynthetic process | Gene Ontology | GO:0046885 | *PRL* | 0.011814 |
| Regulation of hormone metabolic process | Gene Ontology | GO:0032350 | *PRL* | 0.016839 |
| Hormone biosynthetic process | Gene Ontology | GO:0042446 | *PRL* | 0.030133 |
| Body fluid secretion | Gene Ontology | GO:0007589 | *PRL* | 0.031783 |
| Cellular hormone metabolic process | Gene Ontology | GO:0034754 | *PRL* | 0.038361 |
